# Penicillium-Derived Inhibitors of *Plasmodium falciparum* Lactate Dehydrogenase (PfLDH): A Computational Approach for Novel Antimalarial Therapy Development

**DOI:** 10.1155/sci5/8838031

**Published:** 2025-08-04

**Authors:** Imran Sama-ae, Pimthip Muengthongon, Azeezah Tohlaeh, Watcharaphon Rukhachan, Pawika Kiattikul, Fathiyah Samaeng, Aritsara Mitklin, Md. Atiar Rahman, Aman Tedasen, Pattamaporn Kwankaew, Manas Kotepui, Anirut Kepan

**Affiliations:** ^1^Department of Medical Technology, School of Allied Health Sciences, Walailak University, Tha Sala District, Nakhon Si Thammarat, Thailand; ^2^Center of Excellence Research for Melioidosis and Microorganisms (CERMM), Walailak University, Tha Sala District, Nakhon Si Thammarat, Thailand; ^3^Department of Biochemistry and Molecular Biology, University of Chittagong, Chittagong, Bangladesh; ^4^Research Excellence Center for Innovation and Health Products (RECIHP), Walailak University, Nakhon Si Thammarat, Thailand; ^5^Medical Technology Program, Faculty of Science, Nakhon Phanom University, Kham Thao, Nakhon Phanom, Thailand; ^6^Department of Nutrition, Pattani Hospital, Pattani, Thailand

**Keywords:** antimalarial drug development, computer-aided drug discovery (CADD), malaria, penicillium-derived secondary metabolites, *Plasmodium falciparum* lactate dehydrogenase (PfLDH)

## Abstract

Malaria remains a major global health challenge, necessitating the discovery of novel therapeutic agents. This study investigates secondary metabolites from *Penicillium* spp. as potential inhibitors of *Plasmodium falciparum* lactate dehydrogenase (PfLDH), a critical enzyme in the parasite's glycolytic pathway. A curated library of *Penicillium*-derived compounds underwent drug-likeness and toxicity screening, resulting in the identification of 42 viable candidates. Molecular docking simulations revealed three promising compounds—Penicilactone B, Penicillimide, and Penicillisocoumarin A—with binding affinities exceeding those of the positive controls, NADH, and pyruvate. Among these, Penicilactone B exhibited the strongest binding affinity (−8.71 kcal/mol) and the lowest inhibitory constant (414.77 nM). Molecular dynamics simulations confirmed the stability of these compounds within the PfLDH binding pocket over a 200-ns trajectory, with Penicilactone B demonstrating the most stable interactions. Off-target predictions suggested minimal interaction with human lactate dehydrogenase, indicating a potentially favorable safety profile. Penicilactone B emerged as the most promising candidate due to its molecular stability, efficient binding, and favorable solvent interactions. Penicillisocoumarin A also showed potential, supported by its pharmacokinetic properties and safety indicators. These findings highlight the potential of penicillium-derived secondary metabolites as a promising source for novel antimalarial therapies targeting PfLDH. Future research should focus on experimental validation, pharmacokinetic optimization, and efficacy testing against diverse *Plasmodium* strains.

**Summary**

• *Penicillium*-derived metabolites identified as potent PfLDH inhibitors for malaria.
• Penicilactone B shows balanced molecular stability, packing, and solvent interaction.
• Penicillisocoumarin A shows a promising pharmacokinetic profile and safety.
• Molecular dynamics simulations confirm the stability of key compounds over 200 ns.
• Off-target activity prediction confirmed no human lactate dehydrogenase interaction.

## 1. Introduction

Malaria remains a major global health threat, particularly in tropical and subtropical regions. In 2023, an estimated 263 million malaria cases were reported worldwide, with approximately 597,000 deaths—most of which occurred in sub-Saharan Africa [1]. In 2018 alone, estimated 228 million cases and 405,000 deaths were recorded globally, underscoring the persistent burden of this parasitic disease. Malaria is transmitted through the bite of infected *Anopheles* mosquitoes, with *Plasmodium falciparum* (*P. falciparum*) being the most pathogenic of the five *Plasmodium* species infecting humans, capable of causing severe and potentially fatal disease [2].

The World Health Organization (WHO) currently recommends artemisinin-based combination therapies (ACTs) as the first-line treatment for uncomplicated *P. falciparum* malaria [3]. These therapies combine artemisinin derivatives with other antimalarials, such as quinoline compounds, which act by inhibiting hematin polymerization, thereby intoxicating the parasite with ferriprotoporphyrin IX generated during hemoglobin degradation [4]. Other ACT components, such as pyrimethamine and proguanil, inhibit the tetrahydrofolic acid cycle, thus depleting a critical cofactor for DNA synthesis. Despite the efficacy of ACTs, their widespread use has led to the emergence of artemisinin-resistant *P. falciparum* strains, particularly in Southeast Asia and Africa [5, 6]. Resistance is also observed in other malaria species; for instance, *P. vivax*—the most prevalent human malaria parasite worldwide—has demonstrated resistance to chloroquine [7].

Efforts to combat malaria are hindered not only by emerging drug resistance but also by the lack of a fully effective vaccine. Resistance is thought to result from mutations in drug target active sites or biochemical changes in the drug receptor landscape [8]. This highlights the urgent need to identify novel lead compounds and alternative antimalarial agents capable of overcoming resistance. Strategic efforts to discover new molecular targets for rational drug design are therefore critical [9].

One such promising target is *P. falciparum* lactate dehydrogenase (PfLDH), a key enzyme in the parasite's anaerobic glycolytic pathway during the erythrocytic stage. PfLDH catalyzes the conversion of pyruvate to lactate, facilitating the regeneration of NAD^+^ and thereby enabling continuous ATP production in the absence of a complete tricarboxylic acid (TCA) cycle [10, 11]. As *P. falciparum* depends heavily on glycolysis for energy, inhibition of PfLDH disrupts ATP production and ultimately leads to parasite death, making it a valuable drug target [12].

PfLDH differs significantly from human lactate dehydrogenase isozymes (hLDHs) in terms of amino acid composition, substrate specificity, and structural conformation [13]. A distinctive five-residue insertion (DKEWN) in the substrate specificity loop of PfLDH alters the active-site architecture, displaces the nicotinamide ring, and expands the binding pocket volume compared to hLDH [14–16]. These structural distinctions enhance the feasibility of designing selective inhibitors with minimal off-target effects in human hosts [17]. Furthermore, the flexible binding modes within the PfLDH active site provide additional opportunities for structure-based inhibitor design, especially for overcoming emerging drug resistance [18].

In the search for novel therapeutics, secondary metabolites from microorganisms—particularly fungi in the genus *Penicillium*—have shown significant promise. *Penicillium* is renowned for its ability to produce a wide range of bioactive compounds with pharmaceutical applications [19]. Over the years, research on *Penicillium* spp., especially endophytes, has expanded beyond antibiotic production to include metabolites with diverse biological activities [20]. Notable examples include the antibiotic penicillin, discovered by Fleming [21]; the cholesterol-lowering drug compactin (mevastatin) [22, 23]; and the antifungal agent griseofulvin [24]. Additionally, *Penicillium*-derived compounds have demonstrated anti-inflammatory [25–27], antioxidant [28, 29], anticancer [30–33], immunosuppressive [34], antifibrotic [35], antidiabetic [36–38], antiobesity [39, 40], neuroprotective [41], antimicrobial [42, 43], antiviral [44], and antiparasitic [45, 46] activities. These examples underscore the pharmaceutical potential of *Penicillium* as a source of bioactive secondary metabolites.

Traditional drug discovery, while historically successful, is often limited by high costs, extended timelines, and high attrition rates during clinical development [47]. In response, computer-aided drug discovery (CADD) has emerged as a powerful complement to conventional methods. CADD leverages computational tools to efficiently screen large compound libraries, predict target interactions, and optimize pharmacokinetic properties prior to synthesis—thereby accelerating and de-risking the drug development process [48].

Within the CADD framework, virtual screening (VS) plays a central role in prioritizing potential drug candidates based on their predicted binding affinities. Oselusi et al. demonstrated how both structure-based virtual screening (SBVS) and ligand-based virtual screening (LBVS) approaches expedite the discovery of novel antimicrobial agents [49]. Vemula et al. emphasized the integration of VS with artificial intelligence (AI) and machine learning to compress drug development timelines [50]. Gupta et al. further highlighted the synergy between VS and deep learning in early-stage drug discovery applications, including target identification and toxicity prediction [51].

In this context, the present study aims to investigate secondary metabolites derived from *Penicillium* spp. as potential inhibitors of PfLDH. Through a comprehensive computational pipeline—including drug-likeness evaluation, VS, molecular docking, and molecular dynamics (MD) simulations—this study seeks to identify promising PfLDH inhibitors that could serve as new antimalarial agents. This approach not only addresses a critical global health challenge but also contributes to the development of innovative therapeutics to combat drug-resistant malaria.

## 2. Materials and Methods

### 2.1. Penicillium-Derived Natural Compound Library Preparation

To construct the compound library, secondary metabolites derived from *Penicillium* spp. were meticulously selected using the Natural Products Atlas 2.0, an open-access database that provides a comprehensive platform for exploring microbial-derived natural products [52].

### 2.2. Drug-Likeness and Pharmacokinetics Evaluation

The evaluation of the *Penicillium*-sourced compounds for drug-likeness and pharmacokinetic properties was conducted using SwissADME, a freely available web-based tool designed for assessing small molecules' pharmacokinetics, drug-likeness, and medicinal chemistry compliance [53]. The analysis included several widely accepted drug-likeness filters, namely, Lipinski's rule of five (Pfizer) [54], Ghose filter (Amgen) [55], Veber filter (GSK) [56], Egan filter (Pharmacia) [57], and Muegge filter (Bayer) [58], in addition to the Abbott bioavailability score [59] and Pan Assay Interference Compounds (PAINS) alerts [60]. Pharmacokinetic parameters assessed included gastrointestinal (GI) absorption, blood–brain barrier (BBB) permeability, P-glycoprotein (P-gp) substrate status, and inhibition of cytochrome P450 (CYP) enzymes. Inclusion Criteria: All natural compounds from the internal library were included. Exclusion Criteria: Compounds were excluded if they had excessive molecular size (SMILES > 200 characters), poor GI absorption, BBB permeability, P-gp substrate activity, or inhibitory activity against CYP isoforms (CYP1A2, CYP2C19, CYP2C9, CYP2D6, and CYP3A4). Compounds that failed at least one drug-likeness filter, had a bioavailability score < 0.55, or triggered PAINS alerts were also excluded.

### 2.3. Toxicity Prediction

Toxicity prediction was performed using ADMETlab 2.0, a machine learning–based online platform for predicting absorption, distribution, metabolism, excretion, and toxicity (ADMET) profiles [61]. Key toxicity endpoints included inhibition of the human ether-à-go-go–related gene (hERG), human hepatotoxicity (H-HT), drug-induced liver injury (DILI), mutagenicity (Ames test), acute oral toxicity (rat model), FDA maximum recommended daily dose (FDAMDD), carcinogenicity, and respiratory toxicity. Inclusion Criteria: Only compounds that passed drug-likeness and pharmacokinetic screening were considered. Exclusion Criteria: Compounds predicted to be hERG blockers, hepatotoxic, DILI-inducing, mutagenic, acutely toxic, carcinogenic, or respiratory toxic were excluded.

### 2.4. Ligand Structure Preparation

Three-dimensional (3D) structures of the ligands were retrieved from the PubChem database [62]. Preparation steps included the addition of polar hydrogens, assignment of Gasteiger charges, and merging of nonpolar hydrogens using AutoDock Tools (ADT) version 4.2 [63–65]. Ligands were then energy-minimized using Open Babel [66] with the Universal Force Field (UFF) and the Conjugate Gradients algorithm (200 steps, 1-step update frequency, and convergence threshold of 0.1 kcal/mol). Final structures were saved in PDBQT format.

### 2.5. PfLDH Structure Preparation

The crystal structure of PfLDH complexed with NADH and oxamate (PDB ID: 1LDG) was obtained from the Protein Data Bank (https://www.rcsb.org/). The 1.74-Å structure was cleaned using BIOVIA Discovery Studio Visualizer [67] by removing water molecules and co-crystallized ligands. ADT version 4.2 [64, 65] was used to add polar hydrogens, assign Kollman charges, merge nonpolar hydrogens, and define atom types and partial charges [68]. The resulting structure was saved in PDBQT format for docking.

### 2.6. Molecular Docking Simulations

Docking simulations were conducted using AutoDock 4.2. Grid maps of the active site were generated using AutoGrid4 [64], with a spacing of 0.375 Å and grid box dimensions of 60 × 60 × 60 Å centered at *x*: 30.86, *y*: 26.38, and *z*: 37.39. The Lamarckian genetic algorithm (GA) was applied with 50 GA runs per ligand, a population size of 200, 2.5 million energy evaluations, a mutation rate of 0.02, and a crossover rate of 0.80 [69, 70]. Each ligand underwent 10,000 docking trials, repeated five times. Binding energies (Δ*G*_bind_) and inhibitory constants (Ki) were calculated using ADT [64, 65]. NADH and oxamic acid were used as controls and docked with identical settings to validate the protocol by comparing their docked poses with crystallographic conformations.

### 2.7. Protein and Ligand Visualizations

Molecular visualizations were generated using the Mol^∗^ Viewer tool [71], providing high-resolution 3D renderings of protein–ligand interactions and spatial configurations.

### 2.8. Comparison of Docking-Based and Deep Learning–Based Binding Predictions

KDeep, a deep learning–based tool representing a modern quantitative structure–activity relationship (QSAR) approach, was used to validate docking-based predictions. Trained on 3D protein–ligand complexes from the PDBbind database, KDeep predicts binding free energy (Δ*G*_bind_, kcal/mol) and the negative logarithm of IC_50_ (pIC_50_) [72]. Each docked PfLDH–ligand complex was submitted to the KDeep web server (https://playmolecule.org), and results were compared with AutoDock predictions. Two correlation analyses were conducted: Δ*G*_bind_ (AutoDock vs. KDeep) and pKi (converted from AutoDock Ki) vs. pIC_50_ (KDeep).

The pKi was calculated using the following equation:

(1) $$\begin{array}{c} \text{pKi}=-\log_{10}\left(\text{Ki}\left[M\right]\right). \end{array}$$

Pearson correlation coefficients (*r*) and *p* values were computed to evaluate consistency. Statistical analysis was performed using Stata version 16 [73].

### 2.9. MD Simulations

Utilizing the Desmond Schrödinger program [74], MD simulations of the ligand–receptor complexes were performed following a sequential and detailed procedure as outlined in a previous study [75]. This included both the selected hit compounds and NADH, which was employed as a positive control to allow for comparative analysis of structural stability and flexibility. The process began with the precise assignment of hydrogen bonds within each complex, followed by the application of the OPLS4 force field [76]. Each complex was immersed in a TIP3P water model, ensuring a minimum distance of 10 Å from the edge of the simulation box [77]. To replicate physiological conditions, the system was supplemented with a 0.15 M saline solution containing sodium and chloride ions, with chloride ions added in specific quantities to neutralize the net charge of each complex. The simulation environment was maintained at a pressure of 1.01325 bar and a temperature of 310.15 K to mimic physiological conditions [78]. Presimulation preparations involved a dual-phase energy minimization using Maestro's standard protocol, integrating both the steepest descent [79] and LBFGS [80] algorithms to allow the system to reach a local energy minimum. The preparation protocol included an initial (constant number of particles, volume, and temperature [NVT]) ensemble simulation at 10 K using Brownian dynamics, followed by a 12-ps NVT simulation at the same temperature to allow rapid temperature equilibration, all while maintaining restraints on nonhydrogen atoms. Subsequent (constant number of particles, pressure, and temperature [NPT]) ensemble simulations were carried out at varied temperatures and pressures, incorporating distinct relaxation constants and velocity resampling every 1 ps [74]. The principal simulation phase lasted 200 ns under NPT conditions, with simulation data recorded every 200 ps, yielding approximately 1000 frames per system [81]. The equations of motion were integrated using the RESPA integrator [82] with appropriately designated time steps. Temperature and pressure constancy were ensured using the Nose–Hoover thermostat and the Martyna–Tobias–Klein barostat methods, respectively. A cutoff radius of 9.0 Å was employed for calculating both electrostatic and Lennard–Jones interactions. This comprehensive MD setup was applied uniformly across all ligand–receptor systems, including the NADH–PfLDH complex, to ensure consistency. This elaborate and systematic approach was critical in accurately depicting the MD within each ligand–receptor complex. For postsimulation analysis, the Simulation Interaction Diagram tool from the Desmond Schrödinger suite was used [74].

### 2.10. Off-Target Activity Prediction

Potential off-target effects in humans were predicted using SwissTargetPrediction [83], which assesses molecular similarity to known bioactive ligands and infers probable protein targets [84, 85]. This approach provides insights into the likelihood of unintended interactions based on structural resemblance to compounds with known biological targets.

## 3. Results

### 3.1. Penicillium-Derived Natural Compound Library Preparation

A detailed compilation of secondary metabolites derived from *Penicillium* species was established using the Natural Products Atlas 2.0 database. This process led to the identification of 2181 compounds across different *Penicillium* species, underscoring the vast diversity and potential of these fungi for drug discovery applications (Supporting File 1).

### 3.2. Drug-Likeness, Pharmacokinetics, and Toxicity Prediction

A thorough assessment of the drug-likeness and pharmacokinetics of the entire collection of 2181 natural compounds in our in-house library identified 214 compounds that met all the established drug-likeness criteria, as outlined in the Materials and Methods (Supporting File 2). The evaluation included key parameters such as molecular weight, hydrogen bond donors and acceptors, rotatable bond count, topological polar surface area (TPSA), and compliance with medicinal chemistry filters (e.g., Lipinski, Ghose, Veber, Egan, Muegge). Furthermore, specific pharmacokinetic properties related to absorption and metabolism were evaluated using SwissADME. These included GI absorption, BBB permeability, P-gp substrate status, and the potential to inhibit five major CYP isoenzymes – CYP1A2, CYP2C19, CYP2C9, CYP2D6, and CYP3A4. Compounds with low GI absorption, ability to cross the BBB, P-gp substrate activity, or CYP450 inhibition potential were excluded from further analysis. A subsequent toxicity assessment using ADMETlab 2.0 led to the exclusion of 172 compounds due to predicted risks such as hERG inhibition, H-HT, DILI, mutagenicity (Ames test), acute oral toxicity in rats, low FDAMDD, carcinogenicity, or respiratory toxicity (Supporting File 3). After these rigorous evaluations, a final set of 42 compounds remained for molecular docking simulations, offering significant potential for investigating therapeutic interactions and advancing the search for promising drug candidates.

### 3.3. Molecular Docking Simulations

Prior to the molecular docking process, the 3D configurations of the ligands were obtained from the PubChem database [62]. Ten compounds were excluded due to structural deficiencies. Subsequently, molecular docking simulations for 32 natural compounds against PfLDH were conducted using AutoDock 4. Notably, three compounds—Penicilactone B (NPA026699), Penicillimide (NPA029942), and Penicillisocoumarin A (NPA030332)—demonstrated significant binding at the precise locations of the PfLDH active site. These compounds exhibited stronger binding energies compared to the positive controls, NADH (PfLDH's coenzyme), and pyruvate (PfLDH's substrate) (Table 1).

#### 3.3.1. Validation of Docking Protocol

**Figure 1:**
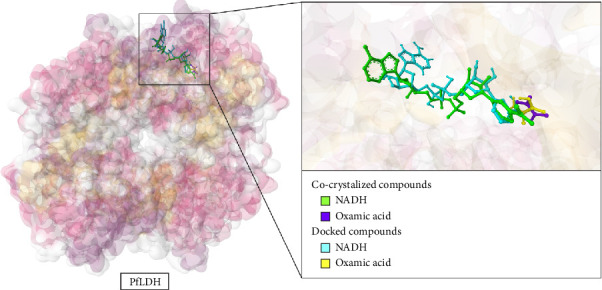
Validation of the docking protocol. Structural alignment of docked and cocrystallized conformations of NADH and oxamic acid within the *Plasmodium falciparum* lactate dehydrogenase (PfLDH) active site confirms the reliability of the docking procedure. Both ligands—retrieved from PubChem and redocked using the same parameters applied to test compounds—accurately occupied their expected binding pockets, closely resembling the conformations observed in the crystal structure (PDB ID: 1LDG).

To assess the reliability of the molecular docking procedure, NADH and oxamic acid—both co-crystallized ligands of PfLDH (PDB ID: 1LDG)—were docked into the enzyme's active site using the same protocol applied to the test compounds. The resulting poses were then compared with their experimentally determined conformations. As illustrated in Figure 1, both ligands docked into the same canonical binding sites as observed in the crystal structure: NADH occupied the NAD-binding pocket, while oxamic acid aligned within the substrate-binding region. This result demonstrates that the docking procedure was capable of directing both ligands into their appropriate binding regions without prior spatial bias. More specifically, the docked pose of NADH exhibited strong alignment with its crystallographic counterpart at the nicotinamide-ribose region, preserving key interactions near the catalytic loop of PfLDH. While the adenine ring displayed a flipped orientation compared to the crystal structure—likely due to the conformational flexibility of NADH and the limitations of rigid-receptor docking—the core binding geometry remained consistent, maintaining critical interaction zones. Similarly, oxamic acid's docked conformation aligned closely with its co-crystallized form in terms of position within the substrate-binding site. Although the docked molecule displayed a flipped orientation compared to the crystal structure—likely a consequence of its molecular symmetry and the energetically similar nature of its termini—the functional groups responsible for anchoring, including the carboxyl and amide moieties, remained within key interacting regions. Overall, the observed overlap between docked and native conformations of both ligands indicates that the docking protocol employed in this study can recapitulate biologically relevant binding modes. Despite minor deviations, especially in the flexible regions of NADH, the approach provides sufficient accuracy and reliability for VS applications targeting PfLDH.

#### 3.3.2. Interactions of Positive Controls With PfLDH

**Figure 2:**
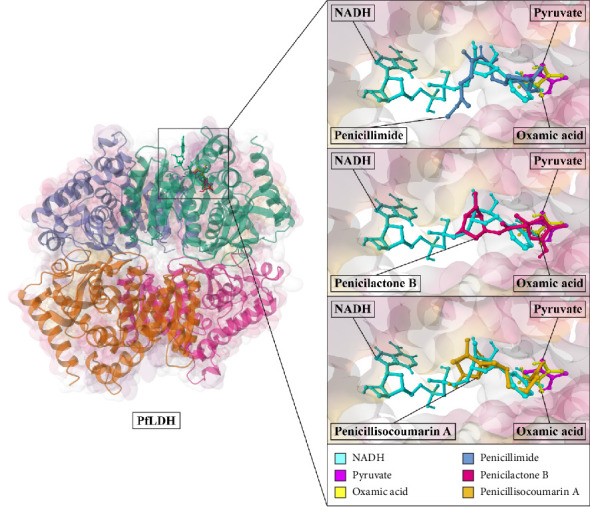
Structural alignment of docked ligands within the PfLDH active site. This multiligand overlay depicts the binding conformations of NADH (cyan), pyruvate (magenta), oxamic acid (yellow), and the three top-scoring penicillium-derived metabolites—Penicillimide (light azure), Penicilactone B (deep pink), and Penicillisocoumarin A (amber)—within the *Plasmodium falciparum* lactate dehydrogenase (PfLDH) binding pocket. The magnified view highlights the spatial alignment of these ligands relative to the native coenzyme and substrate, emphasizing their interactions with key catalytic residues and supporting their potential for competitive inhibition.

**Figure 3:**
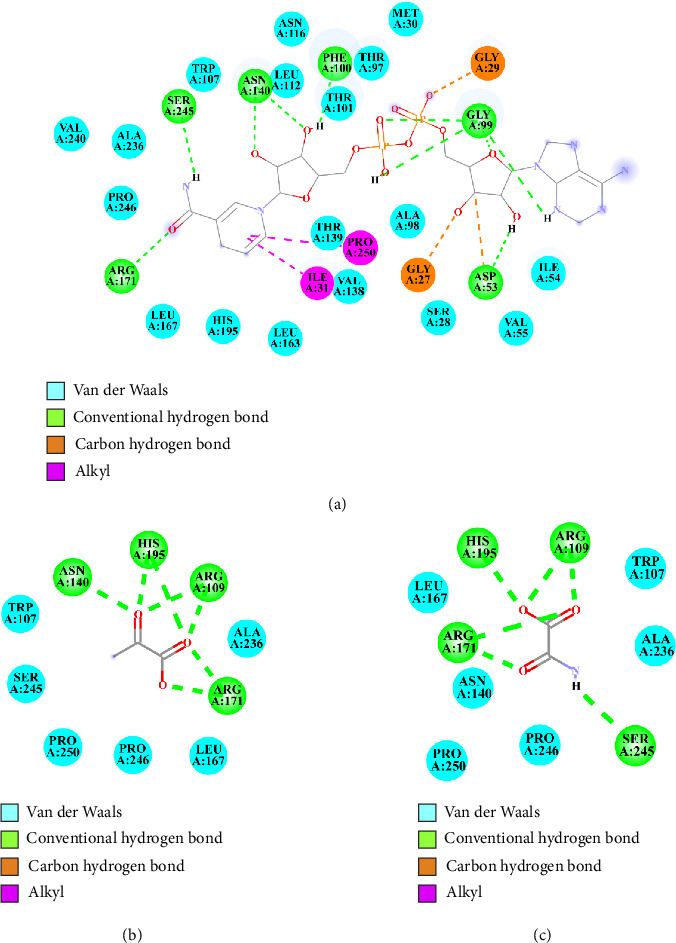
Two-dimensional interaction diagrams illustrating the binding modes of positive controls within the active site of *Plasmodium falciparum* lactate dehydrogenase (PfLDH). The diagrams depict key interactions between the ligands and PfLDH amino acid residues, including van der Waals forces (cyan), conventional hydrogen bonds (green), carbon–hydrogen bonds (orange), and alkyl interactions (magenta). Panels (a–c) represent the following ligands: NADH (a), pyruvate (b), and oxamic acid (c).

NADH (PfLDH's coenzyme) demonstrated substantial binding potential toward PfLDH, with binding energies (Δ*G*_bind_) of −8.2 kcal/mol. Interaction analysis revealed hydrogen bonds with PfLDH residues Gly27, Gly29, Asp53, Gly99, Phe100, Asn140, Arg171, and Ser245, along with van der Waals interactions with residues Ser28, Met30, Ile54, Val55, Thr97, Ala98, Thr101, Trp107, Leu112, Asn116, Val138, Thr139, Leu163, Leu167, His195, Ala236, Val240, and Pro246. Additionally, NADH formed interactions with PfLDH residues Ile31 and Pro250 via alkyl interactions (Figures 2 and 3(a)).

Pyruvate (PfLDH's substrate) displayed binding energies of −5.64 kcal/mol. Interaction analysis revealed hydrogen bonds with PfLDH residues Arg109, Asn140, Arg171, and His195, along with van der Waals interactions with residues Trp107, Leu167, Ala236, Ser245, Pro246, and Pro250 (Figures 2 and 3(b)).

Oxamic acid, a known inhibitor of PfLDH, exhibited a binding energy of −5.25 kcal/mol. Interaction analysis showed that it formed hydrogen bonds with key residues Arg109, Arg171, His195, and Ser245 of PfLDH. Additionally, van der Waals interactions were observed with residues such as Trp107, Asn140, Leu167, Ala236, Pro246, and Pro250 (Figures 2 and 3(c)).

#### 3.3.3. Interactions of Top-Ranked Compounds With PfLDH

**Figure 4:**
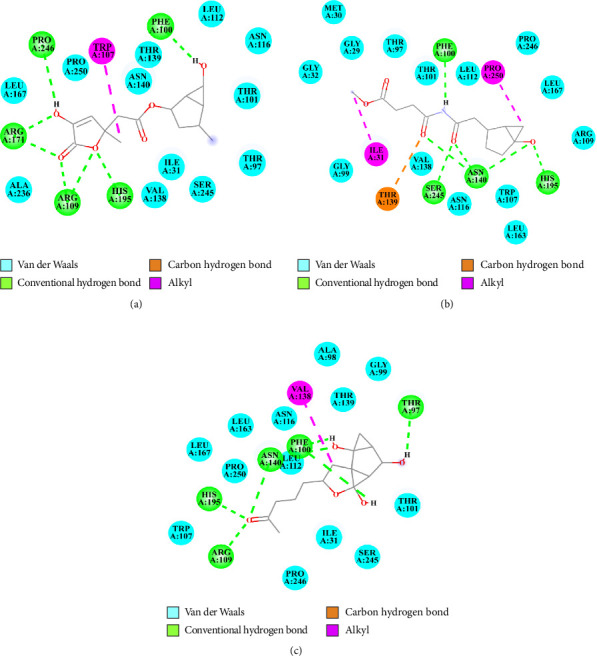
Two-dimensional interaction diagrams illustrating the binding modes of penicillium-derived compounds within the active site of *Plasmodium falciparum* lactate dehydrogenase (PfLDH). The diagrams depict key interactions between the ligands and PfLDH amino acid residues, including van der Waals forces (cyan), conventional hydrogen bonds (green), carbon–hydrogen bonds (orange), and alkyl interactions (magenta). Panels (a–c) represent the following ligands: Penicilactone B (a), Penicillimide (b), and Penicillisocoumarin A (c).

Penicilactone B (NPA026699) exhibited notable affinity for PfLDH with binding energies of −8.71 kcal/mol. The inhibitory constant (Ki) of Penicilactone B toward PfLDH was quantified as 414.77 nM, as presented in Table 1. Interaction analysis revealed hydrogen bonds with PfLDH residues Phe100, Arg109, Arg171, His195, and Pro246, along with van der Waals interactions with residues Ile31, Thr97, Thr101, Leu112, Asn116, Val138, Thr139, Asn140, Leu167, Ala236, Ser245, and Pro250. Additionally, Penicilactone B formed an interaction with PfLDH residue Trp107 via π-alkyl interaction (Figures 2 and 4(a)).

Penicillimide (NPA029942) displayed remarkable binding potential to PfLDH with binding energies (Δ*G*_bind_) of −8.26 kcal/mol. The inhibitory constant of Penicillimide toward PfLDH was quantified as 880.81 nM, as presented in Table 1. Interaction analysis revealed hydrogen bonds with PfLDH residues Phe100, Thr139, Asn140, His195, and Ser245, along with van der Waals interactions with residues Gly29, Met30, Gly32, Thr97, Gly99, Thr101, Trp107, Arg109, Leu112, Asn116, Val138, Leu163, Leu167, Arg171, and Pro246. Additionally, Penicillimide formed interactions with PfLDH residues Ile31, Ala236, Pro246, and Pro250 via alkyl interactions (Figures 2 and 4(b)).

Penicillisocoumarin A (NPA030332) demonstrated binding potential to PfLDH with binding energies (Δ*G*_bind_) of −8.28 kcal/mol. The inhibitory constant of Penicillisocoumarin A toward PfLDH was quantified as 846.72 nM, as presented in Table 1. Interaction analysis revealed hydrogen bonds with PfLDH residues Thr97, Phe100, Arg109, Asn140, and His195, along with van der Waals interactions with residues Ile31, Ala98, Gly99, Thr101, Trp107, Leu112, Asn116, Thr139, Leu163, Leu167, Ser245, Pro246, and Pro250. Additionally, Penicillisocoumarin A formed an interaction with PfLDH residue Val138 via alkyl interactions (Figures 2 and 4(c)).

The molecular docking simulations suggest that Penicilactone B, Penicillimide, and Penicillisocoumarin A have significant binding affinity toward PfLDH, surpassing the binding energies of the positive controls, NADH, and pyruvate. These compounds exhibit multiple interactions with key residues in the active site of PfLDH, indicating their potential as potent inhibitors. The identified hydrogen bonds, van der Waals interactions, and alkyl/π-alkyl interactions are crucial for the stability and efficacy of these potential inhibitors.

### 3.4. Comparison of Docking-Based and Deep Learning–Based Binding Predictions

To further assess the reliability of the docking protocol and support the observed binding affinities, an additional analysis was conducted using KDeep—a deep learning–based predictor trained on the PDBbind dataset. For this purpose, the top-ranked ligands and positive controls were submitted to the KDeep web server. The results included predicted binding free energies (Δ*G*_bind_) and inhibition constants expressed as pIC_50_.

Correlation analyses were performed to compare the binding free energies (Δ*G*_bind_) predicted by AutoDock and KDeep, as well as the pKi values derived from AutoDock's inhibitory constants with the pIC_50_ values predicted by KDeep. The analysis revealed strong positive correlations in both comparisons, with *r* = 0.88 and *p* < 0.001 for Δ*G*_bind_ (AutoDock vs. KDeep), and *r* = 0.87 and *p* < 0.001 for pKi (AutoDock) vs. pIC_50_ (KDeep).

These results demonstrate a high degree of consistency between traditional molecular docking and deep learning–based predictions. For instance, Penicilactone B and NADH exhibited similarly favorable binding affinities and high predicted inhibition constants across both platforms. While some discrepancies in absolute values were noted—likely due to differences in scoring functions and training methodologies—the overall ranking of compounds remained consistent.

**Figure 5:**
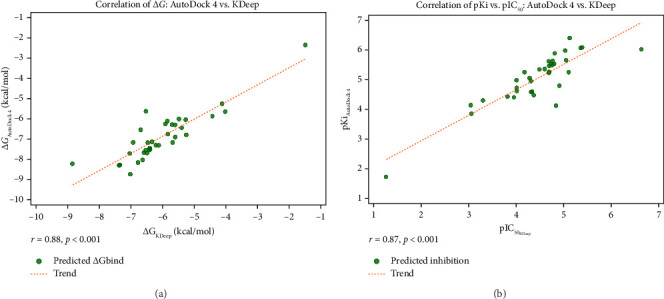
Correlation analysis between AutoDock 4 and KDeep predictions. (a) Scatter plot showing the correlation between predicted binding free energies (Δ*G*, kcal/mol) obtained from AutoDock 4 and KDeep. A strong positive correlation was observed (*r* = 0.88, *p* < 0.001), indicating consistency between the two prediction methods. (b) Scatter plot showing the correlation between predicted inhibition values (pKi from AutoDock 4 vs. pIC_50_ from KDeep). A similarly strong correlation was found (*r* = 0.87, *p* < 0.001), supporting the predictive agreement in estimating inhibitory potential. Each data point (*n* = 35) in both panels represents an individual compound screened in this study. Trend lines are included to illustrate the overall correlation patterns.

These findings underscore the robustness of our VS workflow and offer further validation for the selection of promising lead compounds. Full data are summarized in Supporting File 4, and corresponding scatter plots of correlation analyses are shown in Figure 5.

### 3.5. MD Simulations

The dynamic movements of the docked complexes and their binding stability under conditions mimicking an in vivo environment were further examined through MD simulations conducted over a 200-ns period using the Desmond module of Schrödinger's suite. The results revealed that Penicilactone B, Penicillimide, and Penicillisocoumarin A remained consistently bound within the PfLDH binding pocket throughout the simulation, confirming the complexes' stability under simulated physiological conditions (Supporting Videos 1–3). To validate the simulation framework and provide a benchmark for comparison, NADH—the natural cofactor of PfLDH—was included as a positive control and simulated under identical conditions. The comparative analyses of protein root mean square deviation (P-RMSD) and RMSF between NADH and the selected compounds provided additional insights into the relative stability and flexibility of each ligand–receptor complex.

#### 3.5.1. P-RMSD

**Figure 6:**
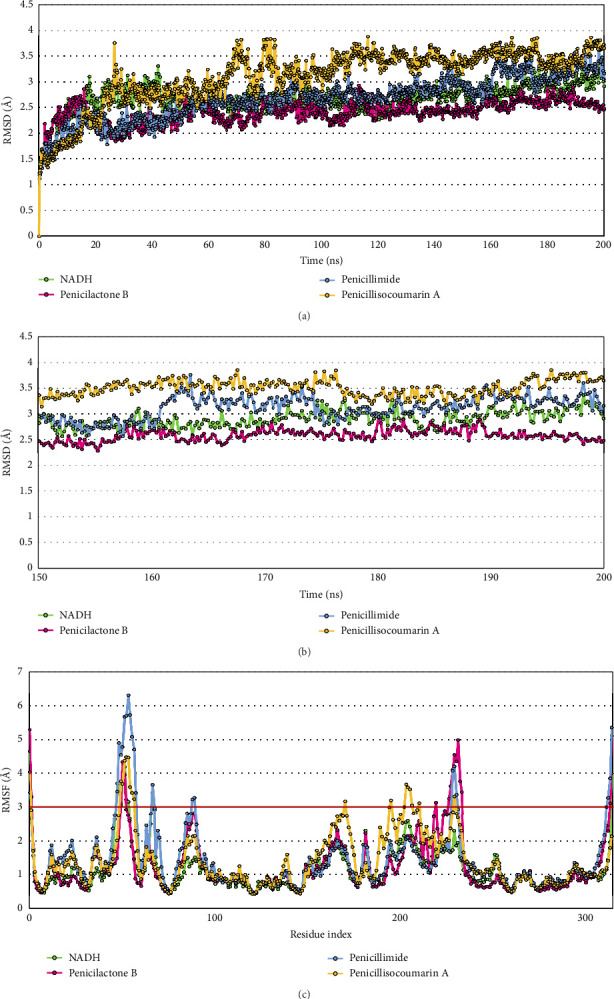
Analysis of root mean square deviation (RMSD) and root mean square fluctuation (RMSF) for PfLDH complexed with three penicillium-derived compounds and the positive control NADH. (a) RMSD analysis (0–200 ns): The root mean square deviation (RMSD) of PfLDH in complex with Penicilactone B (magenta), Penicillimide (blue), Penicillisocoumarin A (orange), and NADH (green) over a 200-ns simulation period. This plot illustrates the overall structural stability of the PfLDH–ligand complexes throughout the entire simulation. (b) RMSD analysis (150–200 ns): A focused RMSD analysis of PfLDH bound to Penicilactone B (magenta), Penicillimide (blue), Penicillisocoumarin A (orange), and NADH (green) during the final 50 ns of the simulation. This section provides a detailed examination of the structural stability of the complexes as they approach the conclusion of the simulation. (c) RMSF analysis: The root mean square fluctuation (RMSF) of PfLDH in complex with Penicilactone B (magenta), Penicillimide (blue), Penicillisocoumarin A (orange), and NADH (green). The RMSF plot reveals the flexibility of individual residues within the PfLDH enzyme when bound to each ligand, identifying regions with high mobility that may influence ligand binding and enzymatic function.

P-RMSD is calculated based on atom selection after aligning all protein frames with the reference frame backbone. Monitoring P-RMSD during the simulation provides valuable insights into the protein's structural conformation.  Figure 6 illustrates the P-RMSD values over the simulation time for the PfLDH protein interacting with three compounds and the positive control NADH. The P-RMSD values for all complexes show a trend in which the deviation increases initially, indicating that the protein undergoes some conformational changes upon binding with the compounds. After this initial change, the P-RMSD appears to plateau, suggesting that the protein–ligand complexes reach a relatively stable conformation. For the NADH–PfLDH complex, the average P-RMSD is approximately 2.681 ± 0.275 Å, with a minimum and maximum of 1.112 Å and 3.366 Å, respectively. For the Penicilactone B–PfLDH complex, the average P-RMSD is approximately 2.408 ± 0.226 Å. The minimum and maximum P-RMSDs are 1.228 Å and 2.932 Å, respectively. For the Penicillimide–PfLDH complex, the average P-RMSD is approximately 2.676 ± 0.420 Å, with a range from 1.174 Å to 3.763 Å. For the Penicillisocoumarin A–PfLDH complex, the average P-RMSD is approximately 3.110 ± 0.538 Å, with a minimum of 1.222 Å and a maximum of 3.873 Å. Overall, the behavior of all complexes, as indicated by the average P-RMSD values, points toward a reasonably stable interaction throughout the simulation, with the expected level of flexibility necessary for biological activity. The most stable complex was observed with Penicilactone B, followed closely by NADH and Penicillimide, while Penicillisocoumarin A exhibited the highest average deviation, though still within an acceptable range. Changes in the order of 1–3 Å are considered acceptable for small, globular proteins. To further interpret these results, it would be beneficial to consider other metrics such as protein root mean square fluctuation (P-RMSF), protein–ligand contacts, ligand torsion profiles, and ligand properties such as ligand RMSD, radius of gyration (rGyr), intramolecular hydrogen bonds (intraHB), molecular surface area (MolSA), solvent-accessible surface area (SASA), and polar surface area (PSA), which can provide additional layers of insight into the stability and behavior of the complex (Figures 6(a) and 6(b)).

#### 3.5.2. P-RMSF

P-RMSF is useful for characterizing local changes along the protein chain. On the P-RMSF plot, peaks indicate areas of the protein that fluctuate the most during the simulation. Typically, the N- and C-terminal tails fluctuate more than other parts of the protein.  Figure 6(c) illustrates the P-RMSF values over the simulation time for the PfLDH protein interacting with three compounds and the positive control NADH. For the NADH–PfLDH complex, the RMSF profile showed moderate flexibility with relatively low peak intensities across most residue positions, reflecting its native compatibility with the PfLDH binding pocket and stable interaction pattern. Peaks were mainly observed at the terminal regions, aligning with expectations for globular proteins. For the Penicilactone B–PfLDH complex, the complex generally shows intermediate flexibility among the three, with significant spikes in certain regions, indicating areas of the protein that undergo more substantial movements relative to the rest of the protein structure. For the Penicillimide–PfLDH complex, the highest overall RMSF values were observed, suggesting that it induces the greatest flexibility within the protein structure. Peaks in RMSF might indicate regions critical for conformational changes or interactions that facilitate its binding or activity. For the Penicillisocoumarin A–PfLDH complex, the complex shows a more moderate level of fluctuation compared to Penicillimide but is generally higher than Penicilactone B. The data show a similar trend of flexibility, with significant peaks indicating mobile regions (Figure 6(c)).

#### 3.5.3. Protein–Ligand Interactions

**Figure 7:**
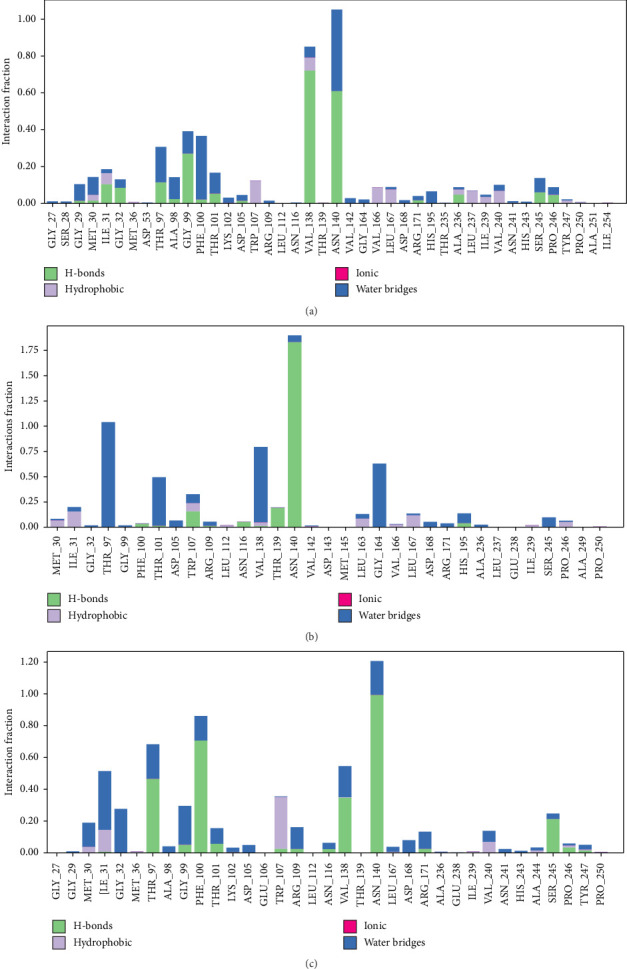
Interaction fractions of PfLDH residues with Penicilactone B (a), Penicillimide (b), and Penicillisocoumarin A (c). The bar graphs represent different types of interactions, including hydrogen bonds (green), hydrophobic interactions (purple), ionic interactions (pink), and water bridges (blue). The *y*-axis indicates the interaction fraction, reflecting the frequency of these interactions throughout the molecular dynamics simulation. Key residues such as ASN140 and VAL138 exhibit prominent interaction profiles, particularly through hydrogen bonds and water bridges, highlighting their importance in ligand binding and stability.

During the simulation, the protein–ligand interactions were closely monitored. Stacked bar charts effectively illustrated that the complexes exhibited various types of interactions, such as hydrogen bonds, hydrophobic interactions, metal coordination, and water bridges (Figure 7). These findings provide comprehensive molecular insights into the interaction dynamics between the ligands Penicilactone B, Penicillimide, and Penicillisocoumarin A with PfLDH, highlighting the critical roles of hydrogen bonding, hydrophobic interactions, metal coordination, and water bridge formations in stabilizing these complexes. This conclusion is further supported by Supporting Videos 1–3, which clearly demonstrate that all three ligands remained consistently within the PfLDH binding pocket and maintained stable interactions throughout the 200-ns simulation period. These observations reinforce the reliability of the initial docked poses and validate the docking protocol used.

#### 3.5.4. Ligand Properties

In the realm of computational drug design, the study of ligand properties plays a pivotal role in predicting the behavior and interaction of potential drug molecules with target proteins. Analyzing these properties allows researchers to infer the efficacy, stability, and overall suitability of ligands as therapeutic agents. Each property provides specific insights that are crucial for optimizing drug design and enhancing the understanding of molecular interactions at the atomic level.

**Figure 8:**
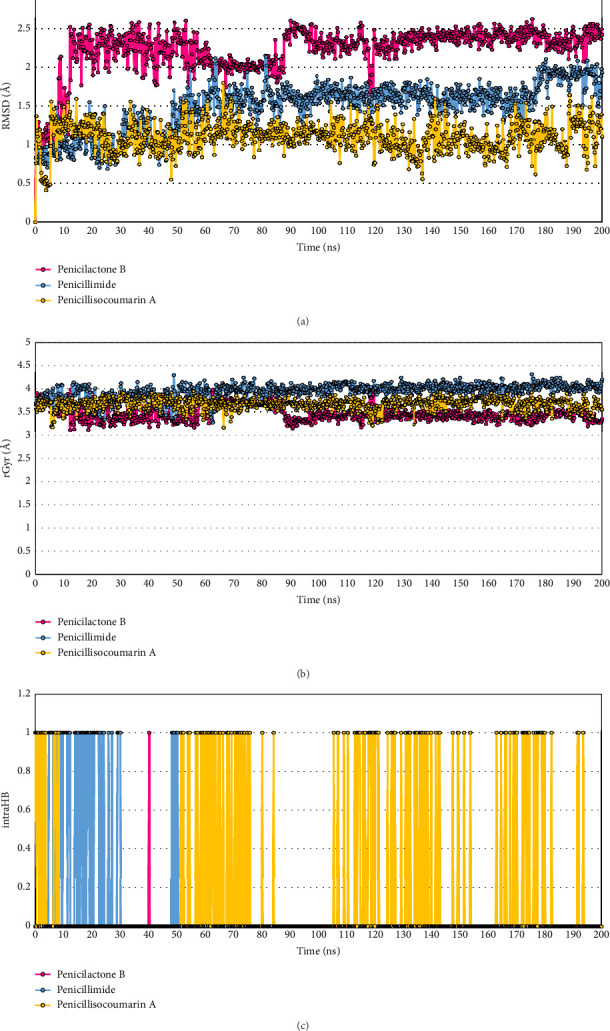
Ligand property analysis of PfLDH complexes with penicillium-derived compounds. (a) Ligand root mean square deviation (L-RMSD): L-RMSD measures the conformational stability of each ligand throughout a 200-ns simulation. Penicilactone B shows a mean L-RMSD of 2.217 Å, Penicillimide has a mean of 1.490 Å, and Penicillisocoumarin A demonstrates the highest stability with a mean L-RMSD of 1.098 Å. (b) Radius of gyration (rGyr): This property quantifies the compactness of the ligands, with Penicillimide exhibiting the largest mean rGyr value of 3.954 Å, indicating a more extended conformation. In contrast, Penicilactone B and Penicillisocoumarin A are more compact, with mean values of 3.484 Å and 3.680 Å, respectively. (c) Intramolecular hydrogen bond (intraHB): The intraHB analysis reveals the internal stability of the ligands, with Penicillisocoumarin A forming the most intramolecular hydrogen bonds (149 occurrences), followed by Penicillimide (81 occurrences) and Penicilactone B (one occurrence).

Ligand root mean square deviation (L-RMSD) measures the consistency of a ligand's conformation throughout a simulation relative to a reference structure, typically its initial conformation. This metric is fundamental in assessing the conformational stability of a ligand under physiological conditions. A low L-RMSD indicates that the ligand maintains a stable conformation, which is often associated with reliable binding and efficacy in drug–receptor interactions. The results illustrated that Penicilactone B had a mean L-RMSD of 2.217 Å with a standard deviation (SD) of 0.314 Å and reached a maximum of 2.621 Å. Penicillimide displayed a mean L-RMSD of 1.490 Å, with an SD of 0.319 Å and a maximum value of 2.151 Å. Penicillisocoumarin A showed a mean L-RMSD of 1.098 Å, an SD of 0.202 Å, and a maximum of 1.799 Å (Figure 8(a)). Although Penicilactone B exhibited higher L-RMSD values compared to the other compounds, this may reflect its conformational flexibility that enables it to adapt and maintain stable interactions within the binding site under dynamic conditions. This flexibility may also enable the ligand to accommodate transient fluctuations within the active site, a characteristic often associated with effective inhibition in physiological environments.

rGyr quantifies the compactness of a molecule in space, serving as a measure of its “extendedness.” This property is critical for understanding how a ligand might fit into the binding pocket of its target protein. Molecules with smaller rGyrs are generally more compact, potentially enhancing their ability to fit into tight binding sites, which can affect their biological activity and solubility. The results illustrated that for Penicilactone B, the mean value was 3.484 Å with a SD of 0.167 Å, a maximum of 4.000 Å, and a minimum of 3.122 Å. For Penicillimide, the mean was 3.954 Å, the SD was 0.154 Å, the maximum reached 4.313 Å, and the minimum was 3.284 Å. Penicillisocoumarin A had a mean of 3.680 Å, an SD of 0.121, a maximum of 3.954 Å, and a minimum of 3.175 Å (Figure 8(b)).

IntraHB provides insights into the internal stability of a ligand. Hydrogen bonds contribute significantly to the structural integrity and rigidity of a molecule. Monitoring the number of intraHB bonds helps predict the flexibility and dynamic behavior of the ligand, which in turn influences its interaction with the target protein. The results illustrated that throughout the simulation time, ranging from 0.00 to 200.20 nanoseconds and across trajectories 0 to 1001, intraHBs were counted as occurring 1 time for Penicilactone B, 81 times for Penicillimide, and 149 times for Penicillisocoumarin A (Figure 8(c)).

**Figure 9:**
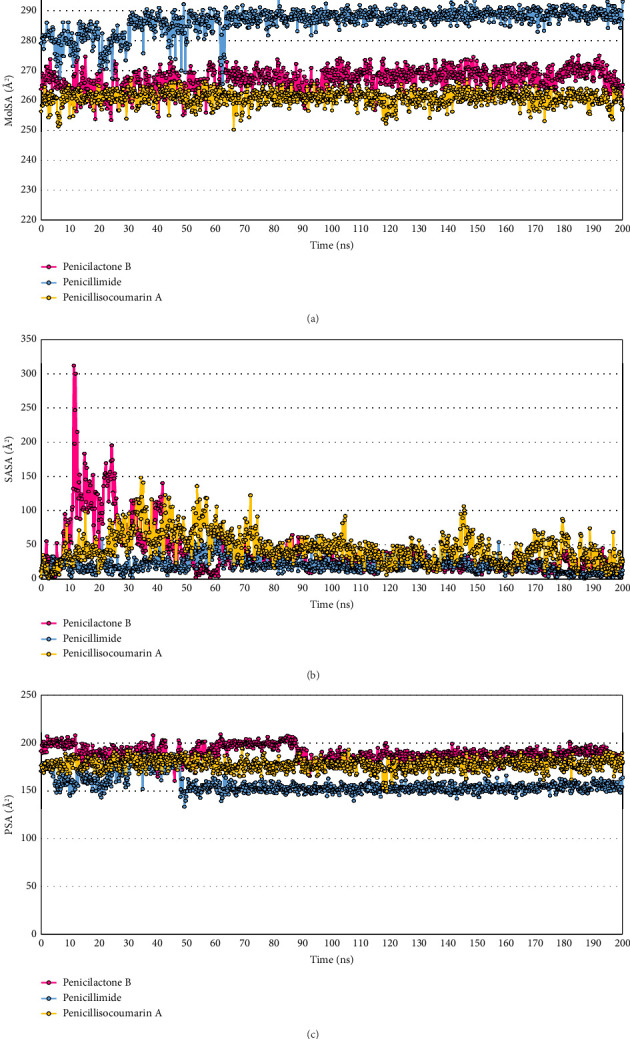
Analysis of ligand properties for *Penicillium*-derived compounds in complex with PfLDH. (a) Molecular surface area (MolSA): Penicillimide exhibits the highest average MolSA at 286.343 Å^2^, indicating a larger surface area for potential interactions, with a maximum value reaching 294.374 Å^2^. Penicilactone B and Penicillisocoumarin A follow with average MolSA values of 266.876 Å^2^ and 260.982 Å^2^, respectively, both showing lower variability. (b) Solvent-accessible surface area (SASA): Penicillisocoumarin A has the highest average SASA at 45.107 Å^2^, suggesting the greatest exposure to solvent. Penicilactone B exhibits the most variability in SASA, with a standard deviation of 35.597 Å^2^ and a maximum of 311.517 Å^2^, indicating significant fluctuations in solvent exposure during the simulation. Penicillimide displays the lowest average SASA at 20.685 Å^2^ and the least variability, suggesting a more consistent interaction with the solvent environment. (c) Polar surface area (PSA): Penicilactone B shows the highest average PSA at 188.776 Å^2^, indicating a strong potential for hydrogen bonding and interaction with biological membranes. Penicillimide, with the lowest average PSA of 157.067 Å^2^, suggests lower polar surface exposure and potentially different transport properties. Penicillisocoumarin A has a moderate average PSA of 177.371 Å^2^, with the least variability among the three ligands.

MolSA and SASA are measures of the total surface area of a molecule. MolSA includes the van der Waals surface area, which is crucial for understanding molecular recognition processes. SASA, on the other hand, measures the surface area accessible to solvent molecules, primarily water, providing valuable information about the solvation properties and likely biological availability of the ligand. The MolSA results for each ligand indicate that Penicillimide has the highest average MolSA at 286.343 Å^2^, followed by Penicilactone B at 266.876 Å^2^, and Penicillisocoumarin A at 260.982 Å^2^. Penicillimide also displayed the highest variability with a SD of 4.183 Å^2^ and a maximum MolSA of 294.374 Å^2^. Penicilactone B and Penicillisocoumarin A showed less variability, with SDs of 3.705 Å^2^ and 2.463 Å^2^, respectively. The minimum MolSA values observed were 253.495 Å^2^ for Penicilactone B, 265.466 Å^2^ for Penicillimide, and 250.342 Å^2^ for Penicillisocoumarin A (Figure 9(a)). The SASA results for each ligand reveal that Penicillisocoumarin A had the highest average SASA at 45.107 Å^2^, followed by Penicilactone B with 37.888 Å^2^, and Penicillimide with the lowest at 20.685 Å^2^. Penicilactone B showed the most variation in its SASA values, with a SD of 35.597 Å^2^ and a maximum SASA of 311.517 Å^2^, indicating significant exposure to solvent during the simulation. Penicillimide had the least variability with a SD of 10.577 Å^2^ and a maximum SASA of 72.516 Å^2^. The minimum SASA values observed were 2.495 Å^2^ for Penicilactone B, 0.686 Å^2^ for Penicillimide, and 1.182 Å^2^ for Penicillisocoumarin A (Figure 9(b)).

Polar surface area (PSA) is particularly relevant in predicting the transport properties of a molecule, such as absorption, permeability, and solubility. This measure reflects the area contributed by oxygen and nitrogen atoms, which are capable of forming hydrogen bonds with water molecules, influencing the ligand's interaction with biological membranes. The PSA results for each ligand are as follows: Penicilactone B had an average PSA of 188.776 Å^2^ with a SD of 7.788 Å^2^, indicating moderate variability. The maximum and minimum PSA values recorded for Penicilactone B were 208.525 Å^2^ and 160.404 Å^2^, respectively. Penicillimide showed an average PSA of 157.067 Å^2^, the lowest among the three ligands, with a higher SD of 9.448 Å^2^, suggesting greater fluctuation in its polar surface exposure. The maximum and minimum PSA values for Penicillimide were 188.749 Å^2^ and 133.688 Å^2^, respectively. Penicillisocoumarin A had an average PSA of 177.371 Å^2^, with the least variability among the ligands, as indicated by a SD of 5.983 Å^2^. Its PSA values ranged from a maximum of 193.924 Å^2^ to a minimum of 149.897 Å^2^. These results highlight the differences in the potential hydrogen bonding capabilities and solubility characteristics of the ligands (Figure 9(c)).

### 3.6. Off-Target Activity Prediction

SwissTargetPrediction is an online tool designed to predict the targets of bioactive small molecules in humans and other vertebrates. This tool is valuable for understanding the molecular mechanisms underlying a given phenotype or bioactivity, rationalizing possible side effects, and predicting off-target interactions of known molecules. Since humans also possess the lactate dehydrogenase enzyme, predicting off-target activity is crucial to prevent potential adverse effects. Using the SwissTargetPrediction platform, the off-target activities of three compounds—Penicilactone B, Penicillimide, and Penicillisocoumarin A—were assessed. The results suggest that none of these compounds are likely to target hLDH. While this prediction indicates a potentially low risk of cross-reactivity with the human enzyme, it should be interpreted with caution, as SwissTargetPrediction is based on structural similarity and does not provide definitive confirmation of biological activity. These results are detailed in Supporting File 5.

## 4. Discussion

Based on the data and insights from the research, several key points were illustrated regarding the potential of Penicilactone B, Penicillimide, and Penicillisocoumarin A as candidates for antimalarial drugs by inhibiting PfLDH.

The first key point concerns binding energies and inhibition constants. Penicilactone B, Penicillimide, and Penicillisocoumarin A demonstrated significant binding affinity toward PfLDH, surpassing the binding energies of the controls, NADH, and pyruvate. This suggests that these compounds are effective at interacting with the PfLDH active site. Penicilactone B exhibited the most potent binding energy at −8.71 kcal/mol and the lowest inhibitory constant at 414.77 nM, indicating strong and efficient inhibition of PfLDH. Meanwhile, Penicillimide and Penicillisocoumarin A also showed competitive inhibition constants, although they were less potent compared to Penicilactone B. These findings align with previous research on PfLDH inhibition. Specifically, studies involving bisquinoline derivatives and chromone-based inhibitors have shown that similar classes of compounds, such as MAQ and BAQ, exhibit strong binding to the PfLDH active site, although they were described as weak inhibitors compared to NADH [17, 86]. Likewise, chromone derivatives demonstrated comparable interaction energies in their docking studies, indicating their potential as PfLDH inhibitors [87]. These findings reinforce the potential of the studied compounds as effective antimalarials targeting PfLDH, with Penicilactone B showing the most potent inhibitory effect based on binding affinity and inhibition constants. In addition to NADH and pyruvate, oxamic acid—a known PfLDH inhibitor—was included in the docking study to provide a benchmark for comparison. Oxamic acid exhibited a binding energy of −5.25 kcal/mol, which is lower than the binding affinities observed for Penicilactone B, Penicillimide, and Penicillisocoumarin A. This suggests that the selected compounds, particularly Penicilactone B, have superior binding potential when compared to this established inhibitor. Notably, oxamic acid formed hydrogen bonds with key active-site residues such as Arg109, Arg171, His195, and Ser245, and exhibited van der Waals interactions with residues like Trp107, Asn140, and Pro246. These interactions partially overlap with those observed in our lead compounds, lending further support to the idea that the identified binding mode is pharmacologically relevant. This comparison reinforces the potential of the screened *Penicillium*-derived compounds as stronger PfLDH inhibitors than oxamic acid, which has previously been validated in structural and functional studies. Moreover, to further validate the docking-based affinity predictions, deep learning–based evaluations were performed using the KDeep platform. Correlation analyses between AutoDock and KDeep results showed a strong agreement, with *r* = 0.88 (*p* < 0.001) for Δ*G*_bind_ and *r* = 0.87 (*p* < 0.001) for pKi versus pIC50. These consistent trends across two distinct computational approaches support the robustness of the binding affinity rankings and reinforce the credibility of the identified lead compounds.

The second key point addresses interaction analysis. All three compounds interacted significantly within the binding site of PfLDH. These interactions included hydrogen bonds and van der Waals interactions, which are essential for strong and stable binding to the enzyme's active site. The variety and types of interactions, such as hydrogen bonds, hydrophobic interactions, and van der Waals forces, suggest that these compounds can robustly anchor themselves within the PfLDH active site. This anchoring potentially interferes with the enzyme's normal function, exhibiting antimalarial activity. These findings align with previous research on quinoline–fluoroproline amide hybrids, which showed that hydrogen bonding and hydrophobic interactions play a critical role in stabilizing the ligand within the PfLDH active site [87]. Similar interaction patterns were reported in studies involving chromone derivatives and other inhibitors, where key residues in the PfLDH enzyme formed hydrogen bonds and van der Waals interactions with the ligands, anchoring them effectively within the enzyme's binding pocket [17]. This corroborates our findings that such interactions are crucial for inhibiting PfLDH activity, reinforcing the potential antimalarial efficacy of these compounds.

The third key point relates to MD simulations. Stability assessments through MD simulations showed that all three compounds maintained interactions within the PfLDH binding pocket over a 200-ns period. This observation affirms the stability of these complexes under physiological-like conditions. The P-RMSD values indicate that the complexes remained relatively stable throughout the simulation period, with Penicilactone B exhibiting the most stable behavior. This stability suggests that Penicilactone B may provide consistent therapeutic effects with minimal conformational changes that could affect drug efficacy. Similar research has demonstrated that MD simulations can effectively predict the stability of ligand–enzyme complexes, with P-RMSD values used as a measure of complex stability. For instance, studies on quinoline-based compounds and other known antimalarials have shown that low P-RMSD values indicate stable binding within the PfLDH active site, confirming the potential of these compounds to act as effective inhibitors [86]. In particular, our observation that Penicilactone B exhibited the most stable behavior mirrors findings from studies on other potent inhibitors. For example, in MD simulations of aminoquinoline derivatives, stable P-RMSD values were correlated with sustained interactions in the active site and suggested better therapeutic potential. The long-term stability of such compounds is critical in maintaining consistent inhibitory activity, as noted in research on other PfLDH inhibitors like amino hydroquinoline-based ligands [87]. In this study, the inclusion of NADH as a positive control provided a valuable benchmark for interpreting simulation results. The P-RMSD and P-RMSF profiles of the NADH–PfLDH complex showed moderate fluctuation with relatively stable behavior, as expected for a native cofactor. When compared to NADH, Penicilactone B exhibited slightly better stability based on average P-RMSD values and comparable P-RMSF trends, highlighting its potential as a strong inhibitor candidate with stability comparable to that of the natural ligand. This comparative observation strengthens the confidence in our simulation setup and suggests that Penicilactone B may function effectively in the PfLDH binding pocket under physiological conditions. Thus, its superior dynamic stability—comparable to the native cofactor NADH—may offer consistent therapeutic effects with minimal conformational changes, supporting its potential as an antimalarial agent.

The fourth key point addresses comparative efficacy. Penicilactone B exhibits molecular interactions similar to those of the natural substrate and cofactor of PfLDH, reinforcing its potential as a competitive inhibitor. Previous studies have reported that inhibitors can bind to the active site of PfLDH in three distinct modes: the pyruvate site, the bridging site between the cofactor and pyruvate, or the adenine binding site [18].

The fifth key point is off-target activity. According to predictions generated by SwissTargetPrediction, these compounds are unlikely to target hLDH, which suggests a lower potential for causing side effects in human hosts. While this prediction is not definitive, it supports the possibility that these compounds may exhibit a favorable safety profile as potential antimalarial agents. Selectivity between PfLDH and hLDH remains a critical factor for minimizing adverse effects in humans, an aspect that has been emphasized in previous studies [88, 89].

The sixth key point relates to ligand properties. L-RMSD is a critical metric in MD simulations, often used to assess the stability of a ligand within a binding site over time. A lower L-RMSD value generally indicates that the ligand maintains a conformation close to its initial binding pose, suggesting a stable and potentially effective interaction with the target protein [90, 91]. In our study, Penicillisocoumarin A exhibited the lowest mean L-RMSD of 1.098 Å, indicating that it maintained a conformation very close to its initial structure, implying a high degree of stability and potentially effective binding. Penicillimide, with a mean L-RMSD of 1.490 Å, also showed high stability, suggesting robust and reliable binding throughout the simulation. Penicilactone B, with a slightly higher mean L-RMSD of 2.217 Å but not over 3 Å, displayed moderate variability, indicating consistent interactions with the binding site, though with slightly more conformational flexibility. rGyr is a crucial parameter in understanding the overall compactness of a molecule, which can directly influence how well a ligand fits into a binding pocket. A lower rGyr value typically suggests a more compact molecular structure, potentially allowing for better accommodation within confined active sites of target proteins [92, 93]. In our study, Penicillimide exhibited the highest mean rGyr of 3.954 Å, suggesting a less compact structure that might struggle to fit into more restricted binding sites, such as the active site of PfLDH. On the other hand, Penicillisocoumarin A and Penicilactone B, with lower mean rGyr values of 3.680 Å and 3.484 Å, respectively, demonstrated more compact structures that could potentially fit better into the PfLDH active site. These findings are consistent with the general understanding that lower rGyr values correlate with better ligand fitting in restricted protein binding sites, enhancing the potential for effective inhibition or modulation of the target protein. IntraHB plays a significant role in determining the stability and flexibility of molecules, which can directly influence their interactions with biological targets. In the context of our study, Penicillimide and Penicillisocoumarin A, which had higher counts of intraHBs (81 and 149, respectively), likely benefit from this increased internal stability, aiding in the maintenance of a favorable conformation for interacting with PfLDH [94, 95]. Conversely, Penicilactone B, with the fewest intraHBs, may exhibit less internal stability but could possess greater flexibility, allowing it to adapt more readily to the enzyme's binding site. This flexibility, while potentially reducing overall stability, might be advantageous in certain dynamic binding scenarios where adaptability is key [96, 97]. MolSA is crucial in drug discovery, indicating a molecule's potential for interaction, especially with protein binding sites. Larger MolSAs typically enable greater interaction with larger binding sites but may reduce specificity. Ertl et al. and Duchowicz and Castro highlighted MolSA's role in predicting drug transport, solubility, and bioavailability, underscoring its importance in drug design [98, 99]. Our study showed that Penicillimide's high MolSA suggests broader interaction but less specificity, while Penicilactone B and Penicillisocoumarin A's lower MolSAs indicate more targeted binding to specific PfLDH residues. SASA is a crucial metric in structural biology and drug design, reflecting the extent to which a molecule is exposed to solvent. Higher SASA values often correlate with increased solubility and bioavailability, as the molecule is more accessible to solvent interactions. This property can significantly influence a molecule's pharmacokinetics and efficacy [100–102]. Our study showed that Penicillisocoumarin A had the highest SASA, indicating it is the most exposed to solvent, potentially enhancing its solubility and bioavailability. Conversely, Penicilactone B and Penicillimide, with lower SASAs, may be less exposed to solvent, suggesting more specific interactions within the PfLDH binding pocket. PSA is a critical factor in drug design, particularly in predicting a compound's transport properties, such as absorption and permeability. PSA is instrumental in assessing how well a compound can interact with biological membranes, influencing its solubility and bioavailability [98, 103, 104]. In line with this, our study found that Penicilactone B, with the highest mean PSA, likely has better solubility and interaction with biological membranes, which is beneficial for oral absorption. Conversely, Penicillimide, which exhibited the lowest PSA, may have limited interaction with aqueous environments, potentially reducing its absorption. Based on the detailed analysis of these ligand properties, Penicilactone B emerges as a strong candidate due to its balance of molecular stability (moderate L-RMSD), efficient packing (moderate rGyr), good solvent interaction (moderate SASA and highest PSA), and sufficient molecular recognition capabilities (moderate MolSA). These properties collectively suggest that Penicilactone B can stably and effectively interact with PfLDH, maintaining good solubility and potentially favorable absorption characteristics. While Penicillisocoumarin A shows the best stability (lowest L-RMSD) and Penicillimide presents high internal rigidity (high intraHB count), the overall balance of properties in Penicilactone B aligns well with the requirements for effective drug–receptor interactions, making it a promising lead for further development as an antimalarial drug.

The seventh key point addresses drug-likeness and pharmacokinetics. Predicting the drug-likeness and pharmacokinetics of Penicilactone B, Penicillimide, and Penicillisocoumarin A based on their chemical and physical properties provides insights into their potential as antimalarial drug candidates (Table 2). Molecular Weight: Molecular weight is crucial in drug design, influencing a compound's oral bioavailability. Compounds with molecular weights under 500 g/mol, as suggested by Lipinski's “Rule of Five”, are generally more suitable for oral administration due to better absorption and permeability. Compounds above this threshold often face issues with membrane permeability [54]. Our study found that Penicilactone B (278.26 g/mol), Penicillimide (299.70 g/mol), and Penicillisocoumarin A (264.27 g/mol) have molecular weights within this ideal range, suggesting they are well-suited for oral bioavailability and promising for further drug development. XLogP3-AA (Partition Coefficient): The partition coefficient is crucial for assessing a compound's lipophilicity, which affects its ability to permeate cell membranes and its pharmacokinetic profile. Lipinski et al. emphasized that moderate lipophilicity, with Log*p* values between 1 and 3, typically enhances membrane permeability and oral bioavailability [54]. In our study, Penicillisocoumarin A, with an XLogP3-AA of 2.1, may have superior membrane permeability compared to Penicilactone B and Penicillimide. This moderate lipophilicity suggests it can better penetrate cell membranes, supporting its potential as an orally bioavailable drug. Hydrogen bond donor and acceptor count: Hydrogen bond donors and acceptors are crucial for determining the interaction of a drug with its biological target, influencing its binding strength and specificity. According to Lipinski et al., the “Rule of Five” suggests that for a compound to have good absorption and permeability, it should generally have no more than five hydrogen bond donors (sum of OH and NH groups) and no more than 10 hydrogen bond acceptors (sum of nitrogen and oxygen atoms) [54]. In our study, all three compounds—Penicilactone B, Penicillimide, and Penicillisocoumarin A—have two hydrogen bond donors and five to six hydrogen bond acceptors, aligning with Lipinski's guidelines. This balance is essential for forming hydrogen bonds with the biological target, such as PfLDH, the enzyme targeted in malaria therapy. These interactions are crucial for the compounds' efficacy, as they contribute to the strength and specificity of binding to the enzyme's active site, enhancing the potential for effective inhibition. Rotatable bond count: the number of rotatable bonds in a molecule is a significant factor in drug design, as it affects the molecule's conformational flexibility and, consequently, its pharmacokinetic properties. Lipinski et al. discussed the importance of molecular flexibility, particularly noting that fewer rotatable bonds generally correlate with better oral bioavailability due to more predictable interactions with biological targets. Molecules with fewer rotatable bonds tend to have more rigid structures, which can lead to more consistent binding to the target site, thus enhancing pharmacokinetic predictability [54]. In our study, Penicilactone B and Penicillisocoumarin A, each with four rotatable bonds, are expected to exhibit less conformational flexibility, resulting in more stable and predictable interactions with their target, PfLDH. Conversely, Penicillimide, with six rotatable bonds, may exhibit greater flexibility, potentially allowing it to explore different conformations within the binding site. While this flexibility can sometimes be advantageous, offering the ligand multiple orientations to fit into the active site, it may also lead to less predictable pharmacokinetic behavior, which can be a disadvantage in drug development. TPSA is a critical determinant of a drug's ability to permeate cell membranes, influencing absorption and distribution. Lower TPSA values are generally associated with better membrane permeability, which is advantageous for drugs targeting intracellular sites. Lipinski et al. discussed the importance of TPSA in drug design, noting that compounds with lower TPSA values tend to have enhanced membrane permeability and, therefore, better oral bioavailability. Their work suggests that TPSA is a reliable predictor of a compound's ability to cross biological membranes, which is essential for effective drug absorption [54]. In our study, Penicillisocoumarin A, with the lowest TPSA (83.8 Å^2^), is likely to exhibit better membrane permeability compared to Penicilactone B (93.1 Å^2^) and Penicillimide (92.7 Å^2^). This lower TPSA value suggests that Penicillisocoumarin A may more effectively reach intracellular targets, like PfLDH, making it a potentially more effective therapeutic agent. To further strengthen the assessment of pharmacokinetic suitability, this study also incorporated predictive analyses of absorption and metabolism. Absorption was evaluated through in silico prediction of GI absorption and P-gp substrate status. All three compounds showed high GI absorption and were not identified as P-gp substrates, indicating favorable oral bioavailability and minimal risk of efflux-related resistance. Additionally, predictions of BBB permeability showed that none of the compounds were likely to penetrate the CNS, which supports a reduced risk of central nervous system side effects—an advantageous feature for antimalarial agents that act peripherally. For metabolism, the inhibitory potential of the compounds toward five key CYP isoenzymes (CYP1A2, CYP2C19, CYP2C9, CYP2D6, and CYP3A4) was evaluated. None of the selected compounds were predicted to inhibit these enzymes, suggesting a low probability of drug–drug interactions and favorable metabolic stability. These insights complement the physicochemical properties previously discussed and collectively support the drug-likeness of the compounds from both chemical and pharmacokinetic perspectives.

The eighth key point is toxicology prediction. The toxicology predictions for Penicilactone B, Penicillimide, and Penicillisocoumarin A indicate promising aspects regarding their safety profiles as potential antimalarial drugs, based on data from ADMETlab 2.0 (Table 3). hERG Blockers: The hERG encodes a potassium channel that is crucial for cardiac repolarization. Blocking this channel can lead to cardiac toxicity, including potentially fatal arrhythmias, making the assessment of hERG blocking potential critical in drug development [105, 106]. In our study, Penicillimide, Penicilactone B, and Penicillisocoumarin A exhibited excellent hERG scores, indicating a very low risk of blocking the hERG channel. While Penicillimide showed a slightly higher value (0.021) compared to Penicilactone B (0.004) and Penicillisocoumarin A (0.01), all values were still within the range considered safe, suggesting a low likelihood of causing cardiac toxicity. This supports their potential as safe therapeutic candidates. H-HT: H-HT is a critical factor in drug development, as it indicates the potential of a compound to cause liver toxicity [107, 108]. In our study, Penicilactone B and Penicillisocoumarin A showed excellent H-HT scores (0.04 and 0.19, respectively), indicating a lower potential for causing liver toxicity. In contrast, Penicillimide scored a medium risk (0.377), suggesting a higher hepatotoxic potential compared to the other compounds. These predictions align with the emphasis on using in silico tools to screen for potential toxicity early in the drug development process, thereby reducing the risk of late-stage failures due to adverse effects on liver health. DILI: DILI is a major concern in drug development, as it can lead to severe liver damage and is a common cause of drug withdrawal from the market [109]. In our study, Penicillisocoumarin A demonstrated an excellent DILI score (0.245), indicating a low risk of causing liver injury. In contrast, Penicilactone B and Penicillimide fell into the medium-risk category, with scores of 0.526 and 0.568, respectively, suggesting a moderate risk of DILI. These findings underscore the importance of DILI prediction in evaluating the safety profile of drug candidates. Ames toxicity: Ames toxicity is a crucial factor in drug development, as it assesses the mutagenic potential of chemical compounds, which can indicate the likelihood of a substance causing genetic mutations and potentially leading to cancer [110]. In our study, Penicillimide and Penicillisocoumarin A showed slightly higher Ames toxicity scores (0.026 and 0.068, respectively) compared to Penicilactone B (0.012). However, all compounds scored excellently, indicating a low potential for mutagenicity and suggesting that they are safe from a genetic toxicity perspective. These excellent scores highlight the compounds' potential as safe candidates for further drug development. Rat oral acute toxicity (ROA toxicity): ROA toxicity is a crucial parameter in drug development, as it helps predict the potential toxic effects of a compound when ingested [111]. In our study, Penicilactone B and Penicillisocoumarin A showed better ROA toxicity profiles (0.019 and 0.027, respectively) compared to Penicillimide (0.12). Although Penicillimide had a higher toxicity score, all compounds were still rated as excellent, indicating a low risk of acute toxicity when administered orally. FDAMDD: FDAMDD is a crucial parameter in drug development, indicating the highest daily dose of a compound that can be administered without causing significant adverse effects [112]. In our study, Penicillimide showed an excellent FDAMDD score (0.016), suggesting a higher permissible daily dose compared to Penicilactone B (0.501) and Penicillisocoumarin A (0.62), which scored in the medium range. This indicates that Penicillimide may be safer at higher doses, whereas the others may require lower daily doses to avoid toxicity. Carcinogenicity: Carcinogenicity is a critical concern in drug development, as it assesses the potential of a compound to cause cancer. In our study, Penicillimide had the best carcinogenicity score (0.031), indicating the lowest potential for cancer risk among the three compounds. Penicilactone B and Penicillisocoumarin A also scored well (0.048 and 0.205, respectively), but Penicillimide's slightly better score suggests it may be the safest in terms of carcinogenicity. All compounds, however, fall within the excellent range, indicating a low overall risk of carcinogenicity. Respiratory toxicity: Respiratory toxicity is a critical concern in drug development, as it assesses the potential of a compound to cause harmful effects on the respiratory system [113]. In our study, Penicillimide exhibited the lowest risk of respiratory toxicity with a score of 0.012, suggesting it is the safest among the three compounds. Penicilactone B and Penicillisocoumarin A also demonstrated low risks, with scores of 0.19 and 0.179, respectively. Despite slight variations, all compounds were rated as excellent, indicating a low overall risk of causing respiratory toxicity. Based on the toxicological prediction data, none of the compounds exhibit serious toxicity concerns, suggesting that all could potentially serve as favorable candidates for further development as antimalarial drugs. Among them, Penicillisocoumarin A might be considered the safest candidate due to its consistently excellent scores across most parameters.

## 5. Conclusions

In conclusion, Penicilactone B, Penicillimide, and Penicillisocoumarin A represent promising leads for the development of new antimalarial drugs targeting PfLDH. Penicilactone B, in particular, stands out due to its potent inhibitory effect, structural stability, and favorable pharmacokinetic and toxicological profiles, making it a strong candidate for further preclinical development.

However, it is important to acknowledge that key challenges remain, particularly concerning bioavailability, metabolic stability, and the potential for off-target effects. While in silico predictions offer valuable initial insights, they may not fully capture the complexity of biological systems.

Therefore, future research should begin with PfLDH enzyme inhibition assays to validate the computational predictions of binding affinity and inhibitory constants. This should be followed by parasite growth inhibition assays using cultured *P. falciparum* strains to assess the compounds' functional efficacy in a biological context. To evaluate safety and selectivity, toxicity assays against human cell lines (e.g., HepG2 or HEK293) should also be conducted. These experiments will help verify not only the biological activity but also the therapeutic window of the compounds.

Moreover, structure–activity relationship (SAR) analysis and potential synergistic effects with current antimalarial drugs should be explored to optimize efficacy and minimize the risk of resistance development.

## Figures and Tables

**Table 1:** Binding affinity and inhibitory constant of penicillium-derived natural compounds against PfLDH.

Compound name	NPAID	PubChem CID	PfLDH (PDB ID:1LDG)	
			Binding affinity kcal/mol)	Inhibitory constant (Ki)
Positive controls				
NADH (PfLDH coenzyme)	N/A	439153	−8.20	979.97 nM
Pyruvate (PfLDH substrate)	N/A	107735	−5.64	73.51 μM
Oxamic acid (PfLDH inhibitor)	N/A	974	−5.25	141.51 μM
Penicillium-derived compounds				
Citreodiol	NPA000361	6442412	−6.78	10.67 μM
Frequentin	NPA002634	23748522	−7.52	3.08 μM
Penicimarin A	NPA003174	71665534	−2.36	18.58 mM
(5S)-2-(3,5-dihydroxyphenyl)-5-hydroxy-3-methylcyclopent-2-en-1-one	NPA004510	122182013	−7.65	2.48 μM
4-Hydroxyphenethyl methyl succinate	NPA005056	44626565	−7.69	2.3 μM
2-(2,4-Dihydroxy-6-methylbenzoyl)-glycerol	NPA007372	123740797	−5.62	75.97 μM
Penicillenol A1	NPA010189	54720258	−6.1	34.0 μM
Palitantin	NPA013500	6438427	−7.67	2.39 μM
Cyclo-(L-Val-L-Leu)	NPA014163	124306170	−6.27	25.55 μM
Epicitreodiol	NPA014250	139587053	−7.28	4.65 μM
Sclerotinin B	NPA015400	12315415	−7.53	3.02 μM
Penicillenol A2	NPA017474	135017477	−6.29	24.63 μM
4-(2′,3′-Dihydroxy-3′-methylbutanoxy)phenethanol	NPA018807	101890757	−6.89	8.97 μM
Decarestrictine G	NPA019135	10104733	−5.86	51.01 μM
Penicimonoterpene	NPA019715	46908201	−8.14	1.08 μM
Penicoffrazin B	NPA026031	146682500	−6	40.15 μM
Penicoffrazin C	NPA026032	146682501	−6.03	38.08 μM
Penicilactone A	NPA026595	146683020	−7.12	6.04 μM
Penicilactone C	NPA026596	146683021	−7.16	5.65 μM
2,3,4-Trihydroxybutyl cinnamate	NPA026626	146683051	−6.43	19.25 μM
Penicilactone B^∗^	NPA026699	146683120	−8.71	414.77 nM
N-(3-acetamidopropyl)-4-hydroxy-3-methoxybenzamide	NPA027718	146683989	−7.15	5.75 μM
N-(3-acetamidopropyl)-3-hydroxy-4-methoxybenzamide	NPA027719	146683990	−6.24	26.65 μM
Penicimenolidyu A	NPA028186	146684380	−8.01	1.34 μM
Penicimenolidyu B	NPA028187	146684381	−7.43	3.58 μM
Penicillimide^∗^	NPA029942	23900002	−8.26	880.81 nM
Penicillisocoumarin A^∗^	NPA030332	156581447	−8.28	846.72 nM
Penicillospirone	NPA030366	137656674	−6.74	11.47 μM
6-Hydroxy-N-acetyl-beta-oxotryptamine	NPA030840	155569890	−7.15	5.77 μM
Methyl 8-hydroxyhexylitaconate	NPA030951	156581949	−7.47	3.32 μM
Ethyl 3(r)-acetamido-3-(4-hydroxyphenyl)propanoate	NPA031790	146681492	−7.29	4.54 μM
4-Hydroxywaraterpol	NPA032728	21635179	−6.53	16.35 μM

^∗^The compounds that have a stronger binding affinity with PfLDH than positive control.

**Table 2:** The chemical and physical properties of the selected compounds Penicilactone B, Penicillimide, and Penicillisocoumarin A (https://pubchem.ncbi.nlm.nih.gov/).

Compound name	Penicilactone B	Penicillimide	Penicillisocoumarin A
PubChem CID	146683120	23900002	156581447
Molecular weight	278.26 g/mol	299.70 g/mol	264.27 g/mol
XLogP3-AA	1.7	0.9	2.1
Hydrogen bond donor count	2	2	2
Hydrogen bond acceptor count	6	5	5
Rotatable bond count	4	6	4
Topological polar surface area	93.1 Å^2^	92.7 Å^2^	83.8 Å^2^

**Table 3:** Toxicology prediction of the selected compounds Penicilactone B, Penicillimide, and Penicillisocoumarin A using ADMETlab 2.0.

Compound name (PubChem CID)	Penicilactone B (146683120)	Penicillimide (23900002)	Penicillisocoumarin A (156581447)
hERG blockers	0.004 (excellent)	0.021 (excellent)	0.01 (excellent)
Human hepatotoxicity (H-HT)	0.04 (excellent)	0.377 (medium)	0.19 (excellent)
Drug-induced liver injury (DILI)	0.526 (medium)	0.568 (medium)	0.245 (excellent)
Ames toxicity	0.012 (excellent)	0.026 (excellent)	0.068 (excellent)
Rat oral acute toxicity (ROA toxicity)	0.019 (excellent)	0.12 (excellent)	0.027 (excellent)
Maximum recommended daily dose (FDAMDD)	0.501 (medium)	0.016 (excellent)	0.62 (medium)
Carcinogenicity	0.048 (excellent)	0.031 (excellent)	0.205 (excellent)
Respiratory toxicity	0.19 (excellent)	0.012 (excellent)	0.179 (excellent)

*Note:* Empirical decision: 0–0.3: excellent; 0.3–0.7: medium; 0.7–1.0: poor.

## Data Availability

The data that support the findings of this study are available in the supporting information of this article.

## Supporting Information

Supporting Information 1 Supporting File 1: Penicillium-derived natural compounds library preparation.

Supporting Information 2 Supporting File 2: Drug-likeness and pharmacokinetics prediction.

Supporting Information 3 Supporting File 3: Toxicity prediction.

Supporting Information 4 Supporting File 4: Comparison of docking-based and deep learning–based binding predictions.

Supporting Information 5 Supporting File 5: Off-target activity prediction.

Supporting Information 6 Supporting Video 1: Molecular dynamics simulations' video of Penicillactone B inside the PfLDH's binding site under in vivo mimic condition (from 1–200 ns).

Supporting Information 7 Supporting Video 2: Molecular dynamics simulations video of Penicillimide inside the PfLDH's binding site under in vivo mimic condition (from 1–200 ns).

Supporting Information 8 Supporting Video 3: Molecular dynamics simulations' video of Penicillisocoumarin A inside the PfLDH's binding site under in vivo mimic condition (from 1–200 ns).
